# Depletion of canonical Wnt signaling components has a neuroprotective effect on midbrain dopaminergic neurons in an MPTP-induced mouse model of Parkinson’s disease

**DOI:** 10.3892/etm.2014.1745

**Published:** 2014-05-28

**Authors:** TING-LI DAI, CHAN ZHANG, FANG PENG, XUE-YUAN NIU, LING HU, QIONG ZHANG, YING HUANG, LING CHEN, LEI ZHANG, WEIDONG ZHU, YU-QIANG DING, NING-NING SONG, MIN LIAO

**Affiliations:** 1 Department of Histology and Embryology, Institute of Neuroscience, Wenzhou Medical University, Wenzhou, Zhejiang 325035, P.R. China; 2 Key Laboratory of Arrhythmias, Ministry of Education of China, East Hospital, Tongji University School of Medicine, Shanghai 200120, P.R. China; 3 Department of Anatomy and Neurobiology, Tongji University School of Medicine, Shanghai 200092, P.R. China

**Keywords:** LRP5, LRP6, β-catenin, MPTP, dopaminergic neuron, Parkinson’s disease

## Abstract

The canonical Wnt signaling pathway is critical for the development of midbrain dopaminergic (DA) neurons, and recent studies have suggested that disruption of this signaling cascade may underlie the pathogenesis of Parkinson’s disease (PD). However, the exact role of the canonical Wnt signaling pathway, including low-density lipoprotein receptor-related protein 5 and 6 (LRP5/6) and β-catenin components, in a mouse model of PD remains unclear. In the present study, the tyrosine hydroxylase (TH)-Cre transgenic mouse line was used to generate mice with the specific knockout of LRP5, LRP6 or β-catenin in DA neurons. Following inactivation of LRP5, LRP6 or β-catenin, TH-immunohistochemical staining was performed. The results indicated that β-catenin is required for the development or maintenance of these neurons; however, LRP5 and LRP6 were found to be dispensable. In 1-methyl-4-phenyl-1,2,3,6-tetrahydropyridine (MPTP)-treated mice, the depletion of LRP5, LRP6 or β-catenin was found to be protective for the midbrain DA neurons to a certain extent. These *in vivo* results provide a novel perspective for the function of the canonical Wnt signaling pathway in a mouse model of PD.

## Introduction

The canonical Wnt signaling pathway is an ancient and conserved signaling cascade, which regulates cell proliferation, fate and behavior in contexts ranging from embryonic development to disease (1). Secreted Wnt proteins exert these effects through a transcription co-activator, β-catenin, which acts as a key mediator of the Wnt signaling pathway. Wnt ligands bind to a receptor complex composed of Frizzled (Fzd) and low-density lipoprotein receptor-related protein 5 or 6 (LRP5/6) (2–5). This complex leads to the accumulation of cytoplasmic β-catenin, which translocates into the nucleus and activates the target genes by binding to members of the TCF/LEF transcription factor family (6). In the absence of Wnt stimulation, the level of cytoplasmic β-catenin remains low as a result of the ubiquitination/proteosome degradation (7).

The canonical Wnt signaling has a critical role in the development of the ventral mesencephalic dopaminergic (DA) neurons, whose selective loss in the substantia nigra (SNc) results in Parkinson’s disease (PD) (8,9). For example, loss of Wnt1 disrupts the development of mesencephalon (10) and Wnt1 expression increases the number of rat midbrain DA neurons *in vitro* (11). Analysis of LRP6 mutant mice has revealed a delay in the onset of DA precursor differentiation (12). In addition, β-catenin controls DA neurogenesis by maintaining the integrity of the neurogenic niche and the progression from progenitors to DA neurons (13). Recently, it has been demonstrated that the key components of the canonical Wnt signaling link to the affected genes in familial PD (14). In particular, the E3 ubiquitin ligase parkin, encoded by PARK2, has been reported to repress β-catenin by inducing β-catenin ubiquitination and degradation (15). Furthermore, Wnt/β-catenin signaling is also involved in certain PD animal models induced by DA neuron-specific toxins, including 6-hydroxydopamine (6-OHDA) (16) and 1-methyl-4-phenyl-1,2,3,6 tetrahydropyridine (MPTP) (17).

However, the exact role of LRP5, LRP6 and β-catenin, key components of canonical Wnt signaling, in adult DA neurons of normal and MPTP-lesioned mice remain unclear. In order to investigate this, in the present study, DA neuron-specific knockout mice with the deletion of LRP5, LRP6 or β-catenin genes were established. These mice, together with wild-type littermates, were subjected to saline or MPTP injection. Using tyrosine hydroxylase (TH)-immunohistochemical staining, the DA neurons in the compact part of the SNc and the density of TH-immunoreactive (TH-ir) axonal terminals in the striatum were quantified, and the neuroprotective effects of the inactivation of LRP5, LRP6 or β-catenin against MPTP exposure in the midbrain DA neurons were thereby investigated.

## Materials and methods

### Genotyping and maintenance of animals

TH-Cre mice were generated and genotyped as previously described (18). For inactivation of LRP5, LRP6 or β-catenin expression in midbrain dopamine-synthesizing neurons, TH-Cre mice were crossed with LRP5^flox/flox^ (19), LRP6^flox/flox^ (20), and β-catenin^flox/flox^ (21) mice, respectively. The TH-Cre;LRP5^flox/+^ offspring were mated with their littermates to generate TH-Cre;LRP5^flox/flox^ mice. In a similar way, TH-Cre;LRP6^flox/flox^ and TH-Cre;β-catenin^flox/flox^ mice were generated. Hereafter these mice are referred to as LRP5 CKO, LRP6 CKO and β-catenin CKO mice, respectively. Animal experiments were reviewed and approved by the Animal Studies Committee at the Tongji University School of Medicine (Shanghai, China).

### MPTP treatment

MPTP (25 mg/kg; Sigma-Aldrich, St. Louis, MO, USA) dissolved in saline was administered via intraperitoneal injection to five-month old wild-type and CKO mice once a day for five consecutive weeks, as reported previously (22–24). Mice used as controls were treated in the same way with injection of an equivalent volume of saline instead of MPTP.

### Immunohistochemistry

Mice were deeply anaesthetized with sodium pentobarbital (100 mg/kg body weight), and perfused transcardially with 0.01 M phosphate-buffered saline (PBS; pH 7.4), followed by 4% paraformaldehyde in 0.1 M phosphate buffer (pH 7.4) following the five weeks of injections. Brains were dissected out, post-fixed overnight and cryoprotected with 30% sucrose in PBS overnight at 4°C. Transverse sections (40 μm) were cut on a cryostat (CM1950; Leica, Mannheim, Germany), and every sixth section was collected as one set of serial sections that were processed for TH immunohistochemistry. Brain sections were pretreated with citrate buffer (pH 6.0) at 95°C for 6 min and then incubated overnight at 4°C with mouse anti-TH antibody (1:40,000; Sigma-Aldrich) diluted in PBS containing 0.3% Triton X-100 and 1% bovine serum albumin (BSA). Sections were then incubated with biotinylated horse anti-mouse immunoglobulin G (1:500; Vector Laboratories, Burlingame, CA, USA) in the aforementioned PBS/Triton X-100/BSA buffer for 3 h, and then for 1 h with an avidin-biotin-peroxidase complex (1:200; Vector Laboratories) in PBS at room temperature. TH immunoreactivity was visualized using 0.05% 3,3′-diaminobenzidine, as well as 0.04% hydrogen peroxide in PBS.

### Microscopy and imaging

Images were captured on a microscope (Eclipse 80i; Nikon, Tokyo, Japan) equipped with a digital camera (DS-Ri1; Nikon). All images were imported into Photoshop software (Adobe Systems, Inc., San Jose, CA, USA) and minor adjustments to the contrast and brightness were applied if necessary.

### Cell counting

The number of TH-positive neurons was counted in every sixth 40-μm-thick transverse section. TH-positive cells in the SNc were counted for quantitative comparison between wild-type and CKO mice. Statistical significance was determined using a one-way analysis of variance (ANOVA), followed by a post-hoc least significant difference (LSD) test. Error bars represent the standard error of the mean (SEM) and P<0.05 was considered to indicate a statistically significant difference.

### Striatal densitometry

The density of striatal DA terminals was measured as the optical density of the striatal TH-ir using ImageJ software (National Institutes of Health, Bethesda, MA, USA). Four sections were randomly selected from those containing the striatum at the approximate level of Bregma −1.10 to 0.22 mm (25), and the optical density in the central striatum of each section was measured on each side. In each section, the optical densities were corrected by subtraction of background staining in the corpus callosum. Statistical analysis was performed using a one-way ANOVA with post hoc LSD test. Data are presented as the mean ± SEM and P<0.05 was considered to indicate a statistically significant difference.

## Results

### LRP5 CKO

**Figure 1:**
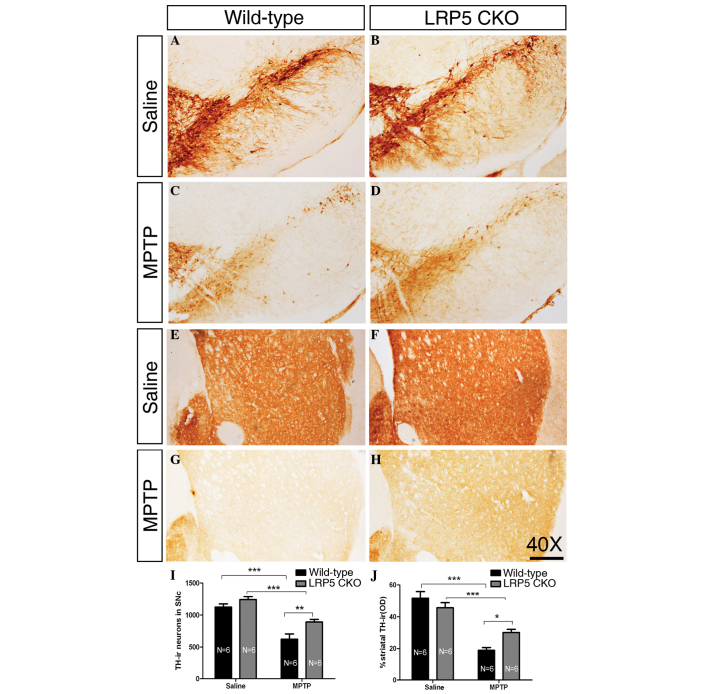
LRP5 deletion attenuates MPTP toxicity. (A–D) Representative images showing TH-ir neurons of the SNc in wild-type and LRP5 CKO mice treated with saline or MPTP. (E–H) Representative images of TH-ir axonal terminals in the striatum of wild-type and LRP5 CKO mice treated with saline or MPTP. (I) Quantification of TH-ir cells in the SNc in the wild-type and LRP5 CKO mice treated with saline or MPTP. A significant difference is found between the two genotypes following MPTP treatment. (J) Statistical data of the optical density of TH-ir striatal terminals in the wild-type and LRP5 CKO mice following treatment with saline or MPTP. There is a significant difference between the two genotypes following MPTP injection. Sample sizes are indicated. Error bars represent the standard error of the mean and asterisks indicate significant differences (^*^P<0.05, ^**^P<0.01 and ^***^P<0.001). Scale bar, 250 μm; magnification, ×40. LRP5, lipoprotein receptor-related protein 5; MPTP, 1-methyl-4-phenyl-1,2,3,6-tetrahydropyridine; TH-ir, tyrosine hydroxylase-immunoreactive; SNc, substantia nigra.

In wild-type and LRP5 CKO mice treated with saline, no significant difference in the number of DA neurons in the SNc was observed (Fig. 1A, B and I), and the density of TH-ir axonal terminals in the striatum was comparable between the two genotypes (Fig. 1E, F and J). Following MPTP administration once per day for five weeks, the number of nigral TH neurons in wild-type mice was reduced to ~55.3% of that in wild-type mice treated with saline (Fig. 1A, C and J). By contrast, although the MPTP treatment also resulted in a marked reduction in the number of TH-ir neurons in the SNc of LRP5 CKO mice compared with that in the saline-treated LRP5 CKO mice, an increase in the number of TH-ir neurons was observed compared with that in the MPTP-treated wild-type mice. In the MPTP-treated LRP5 CKO mice, the TH-ir neuronal number was decreased to ~73.9% of that in LRP5 CKO mice treated with saline (Fig. 1B, D and I). Consistently, while the MPTP treatment led to significant reductions in the density of striatal TH-ir axon terminals in wild-type mice and LRP5 CKO mice, there was a greater density of TH-ir axon terminals present in the striatum of the MPTP-treated LRP5 CKO mice (Fig. 1G, H and J). These results indicate that in the absence of LRP5 expression, a greater number of midbrain DA neurons survived following exposure to MPTP.

### LRP6 CKO

**Figure 2:**
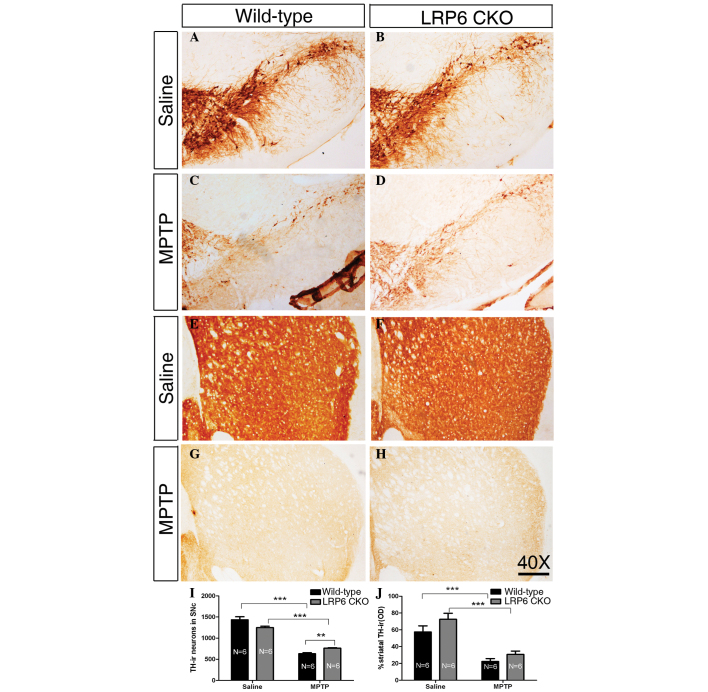
LRP6 CKO mice are resistant against MPTP toxicity to a certain extent. (A–D) Representative images of nigral TH-ir neurons in wild-type and LRP6 CKO mice subjected to saline or MPTP injection. (E–H) Representative images of striatal TH-ir axonal terminals of wild-type and LRP6 CKO mice treated with saline or MPTP. (I) Quantitation of TH-ir neurons in the SNc of wild-type and LRP6 CKO mice treated with saline or MPTP injection. There is a significant difference between the two genotypes following MPTP treatment. (J) Statistical data of the optical density of TH-ir striatal terminals in the wild-type and LRP6 CKO mice treated with saline or MPTP injection. No significant difference between the two genotypes following MPTP treatment is detected. Sample sizes are indicated. Error bars represent the standard of the mean and asterisks indicate significant differences (^**^P<0.01 and ^***^P<0.001). Scale bar, 250 μm; magnification, ×40. LRP6, lipoprotein receptor-related protein 6; MPTP, 1-methyl-4-phenyl-1,2,3,6-tetrahydropyridine; TH-ir, tyrosine hydroxylase-immunoreactive; SNc, substantia nigra.

In wild-type and LRP6 CKO mice treated with saline injections for five weeks, the TH-ir neurons in the SNc were intensely immunostained, and TH-ir axons were densely and evenly distributed throughout the striatum, without a detectable difference between the two genotypes (Fig. 2A, B, E, F, I and J), indicating that the midbrain DA neurons and their nigrostriatal projection are morphologically normal in the absence of LRP6 expression in adulthood. MPTP administration induced a marked loss of TH-ir neurons in the SNc of wild-type mice, and this reduction was significantly higher compared with that in the MPTP-treated LRP6 CKO mice (Fig. 2C, D and I), reflecting the possibility that the loss of LRP6 is beneficial for the midbrain DA neurons exposed to MPTP. However, the nigrostriatal projection shown by TH-ir axons in the striatum was similar between wild-type and LRP6 CKO mice in terms of the density following the MPTP treatment (Fig. 2G, H and J). Thus, MPTP treatment leads to a decreased loss of midbrain DA neurons in LRP6 CKO mice compared with that in wild-type mice; however, this reduction is not reflected by the density of TH-ir striatal axons.

### β-catenin CKO

**Figure 3:**
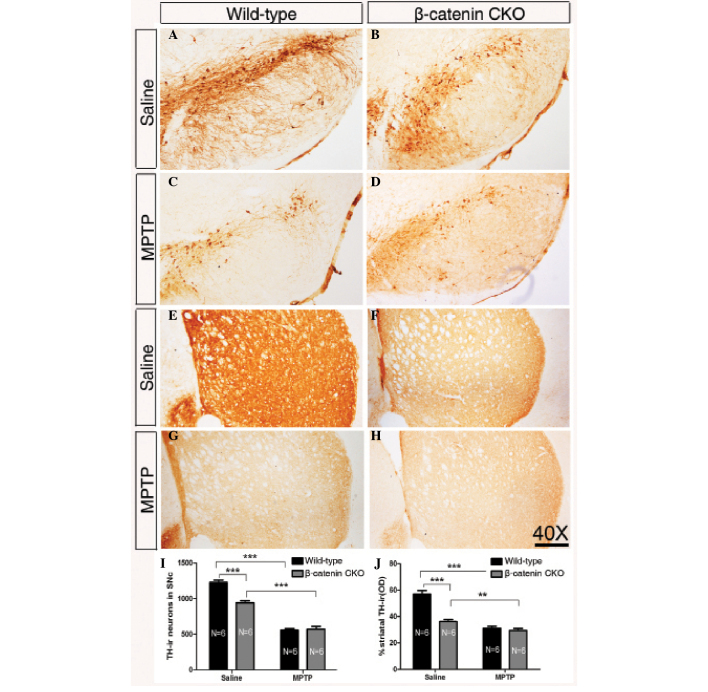
Inactivation of β-catenin reduces the number of DA neurons and protects them from exposure to MPTP to a cetain extent. (A–D) Representative images showing nigral TH-ir cells in wild-type and β-catenin CKO mice treated with saline or MPTP injection. (E–H) Representative images of striatal TH-ir axonal terminals of wild-type and β-catenin CKO mice treated with saline or MPTP. (I) Quantification of TH-ir neurons in the SNc of wild-type and β-catenin CKO mice following saline or MPTP injection. The number of TH-ir neurons is decreased in β-catenin CKO mice compared with that in wild-type mice folowing treatment with saline. Following MPTP treatment, no significant difference is observed between the two genotypes. (J) Statistical data of the optical density of TH-ir striatal terminals in the wild-type and β-catenin CKO mice treated with saline or MPTP. A similar significant difference between the two genotypes following saline treatment is detected, while no change is found following MPTP treatment. Sample sizes are indicated. Error bars represent the standard of the mean and asterisks indicate significant differences (^**^P<0.01 and ^***^P<0.001). Scale bar: 250 μm; magnification, ×40. DA, dopaminergic; MPTP, 1-methyl-4-phenyl-1,2,3,6-tetrahydropyridine; TH-ir, tyrosine hydroxylase-immunoreactive; SNc, substantia nigra.

In saline-treated mice, the number of TH-ir neurons in the SNc was lower in the β-catenin CKO mice relative to that in the wild-type mice, with an approximate ratio of 76.7% (Fig. 3A, B and I; P<0.001). Consistently, the density of TH-ir axons in the striatum was higher in the wild-type mice compared with that in the β-catenin CKO mice (Fig. 3E, F and J). The decreased number of TH-ir neurons in adult β-catenin CKO mice suggests that β-catenin may be required in the development and/or maintenance of the midbrain DA neurons.

Following the five-week MPTP treatment, although the TH-ir neurons in the SNc were significantly reduced quantitatively in wild-type and β-catenin CKO mice, no marked difference was observed between the two genotypes in terms of the number of TH-ir neurons in the SNc and the density of the TH-ir axons in the striatum (Fig. 3C, D and G–J). Considering the fact that fewer TH-ir neurons remained in β-catenin CKO mice than in the wild-type mice following saline treatment, these results suggest that midbrain DA neurons lacking β-catenin expression appear to be resistant to MPTP toxicity to a certain extent.

## Discussion

The canonical Wnt signaling pathway is critical for many cellular processes. LRP5/6, co-receptors for Wnt ligands, are highly homologous proteins with key functions in this signaling pathway, including development, as well as disease (26,27). However, the role of LRP5/6 in the development of midbrain DA neurons and their association with PD remain unclear. In addition, the function of β-catenin, the key mediator of canonical Wnt signaling, is not yet clearly understood in mouse PD models. Therefore, in the present study the expression of LRP5, LRP6 or β-catenin was inactivated in the midbrain DA neurons of the LRP6, LRP6 or β-catenin CKO mice, and the alterations in the numbers of TH-ir neurons in the SNc and striatum of the MPTP-PD model were then investigated.

LRP5/6 have previously been demonstrated to be critical co-receptors by binding to Wnt-Fzd to form a trimeric complex (28); however, the analysis of genetically engineered mice has revealed their different functions during embryonic development (27). Generally, the LRP6 loss-of-function phenotypes are more severe compared with the LRP5 loss-of-function phenotypes (5), indicating that LRP6 has a more crucial role during embryogenesis. In accordance with this, in LRP6 mutant mice a marked reduction in the number of TH-positive neurons and a defect in midbrain morphogenesis were observed at embryonic day (E) 11.5 (12). However, in the present study, no change in the number of DA neurons in saline-treated LRP6 CKO mice (Fig. 2A, B, E, F, I and J), as well as in LRP5 CKO mice (Fig. 1A, B, E, F, I and J), compared with that in saline-treated wild-type mice, was observed. Given that a global knockout mouse model was used in the previous study, this may suggest that i) loss of LRP6 in cell types other than in DA neurons of the midbrain causes the developmental delay of DA neurons, while the selective knockout of LRP6 in midbrain DA neurons has no apparent impact; and ii) the Cre recombinase in TH-Cre mice is firstly expressed in postmitotic midbrain DA neurons after E12, so the initial process of DA neurogenesis is not interrupted in the LRP6 CKO mice.

β-catenin is an obligate component of the Wnt signaling cascade, and recent studies have shown that it is critical for midbrain DA neuron specification and neurogenesis (29). In particular, β-catenin loss-of-function experiments showed that key DA progenitor genes, including Otx2, Lmx1a, Msx1 and Ngn2 are downregulated and fewer DA neurons are generated (13,30). In accordance with these results, in the present study, it was found that there is a reduction in the number of midbrain DA neurons and striatal DA terminals in β-catenin CKO mice compared with that in wild-type mice when injected with saline (Fig. 3A, B, E, F, I and J), suggesting a cell-autonomous function of β-catenin in DA neurons.

Symptoms of PD may be induced in mouse by DA neuron-specific toxins, including 6-OHDA, rotenone and MPTP (31). In the present study, chronic MPTP treatment was performed, which has previously been shown to be more effective compared with acute or sub-acute protocols (32). In wild-type mice, MPTP led to the loss of approximately half of the nigral DA neurons and the striatal DA terminals, compared with those in the saline-injected mice (Fig. 1–3), indicating that the mouse model for PD was successfully generated.

Following MPTP treatment for five weeks, the numbers of TH-ir DA neurons in the midbrain and of TH-ir axonal terminals in the striatum were decreased in LRP5/6 CKO mice in a similar manner to that in control mice; however, the number of surviving DA cells in the CKO mice was increased compared with that in the wild-type mice (Figs. 1A, B and I and 2A, B and I). These data suggest that specific ablation of LRP5/6 in midbrain DA neurons has a neuroprotective role in MPTP-treated mice. Although the DA neurons were decreased in number in the β-catenin CKO mice without MPTP exposure, the numbers of surviving DA neurons were comparable between wild-type and β-catenin CKO mice following chronic MPTP injection (Fig. 1A, B and I), indicating a similar protective effect in β-catenin depleted mice and LRP5/6 CKO mice. However, recent studies have suggested that canonical Wnt signaling contributes to the protection of midbrain DA neurons (14). For example, the Wnt signaling antagonist Dickkopf-1 aggravates the DA neuron damage of SNc in 6-OHDA-lesioned rats (16) and counteracts astrocyte-induced neuroprotection against MPTP toxicity in primary mesencephalic astrocyte-neuron cultures (17). In addition, an *in vitro* study showed that exogenous Wnt1 protects primary mesencephalic DA neurons against cell death induced by 6-OHDA or MPTP, and this neuroprotection is abolished by the knockdown of β-catenin or Fzd (33). These results are based on *in vitro* cultures or pharmacological studies *in vivo*; thus, a genetically engineered mouse model involved in Wnt signaling is lacking. In the present study, the Cre-loxP strategy (34) was used to conditionally knockout key components of the canonical Wnt signaling pathway and MPTP-induced PD mouse model, which may be more reliable and reflect the physiological condition. However, inconsistent results were obtained (16,17,33). Therefore, further investigations are required to clearly elucidate the role of the canonical Wnt signaling pathway in PD.

In conclusion, in the present study, LRP5, LRP6 or β-catenin were selectively knocked out in the midbrain TH-positive neurons. The results indicated that the loss of β-catenin affects the survival and/or maintenance of the midbrain DA neurons, while the loss of LRP5/6 does not. When exposed to MPTP, the LRP5, LRP6 or β-catenin CKO mice showed that the depletion of these Wnt signaling components has a neuroprotective effect on the midbrain DA neurons. These data provide a novel perspective for the role of canonical Wnt signaling components in the pathogenesis of PD.
